# Cancerous Inhibitor of Protein Phosphatase 2A (CIP2A): Could It Be a Promising Biomarker and Therapeutic Target in Parkinson’s Disease?

**DOI:** 10.1007/s12035-021-02670-w

**Published:** 2022-01-05

**Authors:** Sijia Yin, Chao Han, Yun Xia, Fang Wan, Junjie Hu, Liang Kou, Yadi Sun, Jiawei Wu, Yunna Li, Qiulu Zhou, Nian Xiong, Jinsha Huang, Tao Wang

**Affiliations:** 1 Department of Neurology, Union Hospital, Tongji Medical College, Huazhong University of Science and Technology, 1277 Jiefang Avenue, Wuhan, 430022 People’s Republic of China; 2 Department of Neurology, The First Affiliated Hospital of USTC, Division of Life Sciences and Medicine, University of Science and Technology of China, Hefei, Anhui 230001 People’s Republic of China

**Keywords:** Parkinson’s disease, Biomarker, PP2A, CIP2A, Alpha-synuclein

## Abstract

Parkinson’s disease (PD) is an incurable neurodegenerative disease characterized by aggregation of pathological alpha-synuclein (α-syn) and loss of dopaminergic neuron in the substantia nigra. Inhibition of phosphorylation of the α-syn has been shown to mediate alleviation of PD-related pathology. Protein phosphatase 2A (PP2A), an important serine/threonine phosphatase, plays an essential role in catalyzing dephosphorylation of the α-syn. Here, we identified and validated cancerous inhibitor of PP2A (CIP2A), as a potential diagnostic biomarker for PD. Our data showed that plasma CIP2A concentrations in PD patients were significantly lower compared to age- and sex-matched controls, 1.721 (1.435–2.428) ng/ml vs 3.051(2.36–5.475) ng/ml, *p* < 0.0001. The area under the curve of the plasma CIP2A in distinguishing PD from the age- and sex-matched controls was 0.776. In addition, we evaluated the role of CIP2A in PD-related pathogenesis in PD cellular and MPTP-induced mouse model. The results demonstrated that CIP2A is upregulated in PD cellular and MPTP-induced mouse models. Besides, suppression of the CIP2A expression alleviates rotenone induced aggregation of the α-syn as well as phosphorylation of the α-syn in SH-SY5Y cells, which is associated with increased PP2A activity. Taken together, our data demonstrated that CIP2A plays an essential role in the mechanisms related to PD development and might be a novel PD biomarker.

## Introduction

Currently, diagnosis of Parkinson’s disease (PD) is mainly dependent on clinical symptoms. Since the clinical symptoms are hardly definitive, there is need for identification of appropriate biomarkers to aid in the diagnosis of PD. Besides, a comprehensive understanding of the pathogenesis of PD would aid implementation and development of neuroprotective therapy, especially early stage, where the clinical features cannot be relied upon. Thus, an adequate biomarker would help personalize protective treatment protocols and care.

PD is characterized by the loss of dopaminergic neurons in the substantia nigra (SN) [1] and the presence of Lewy bodies (LBs) in the remaining dopaminergic neurons. Alpha-synuclein (α-syn) is the main protein in the LBs, and 90% of the deposited α-syn is phosphorylated at serine 129 (Ser 129), while only 4% is phosphorylated in the normal brain [2]. Studies on familial and sporadic PD patients have shown that phosphorylation of Ser129 (p-α-syn) contributes to the formation of the LBs [3, 4]. Other studies have shown that p-α-syn is a specific pathological event and closely associated with the pathology of PD. Protein phosphatase 2A (PP2A) is the main phosphatase of the p-α-syn [5, 6]; suppression of its activity mediates the hyperphosphorylation of α-syn. Thus, targeting PP2A might have therapeutic potential, which could reduce the phosphorylation of α-syn in PD and slow down the accumulation of pathological proteins [7, 8].

CIP2A, an endogenous inhibitor of PP2A, is an oncoprotein encoded by the KIAA1524 gene [9]. Previous studies have demonstrated that CIP2A is overexpressed in various human malignancies, promotes the initiation and proliferation of cancer cells as well as acting as a prognostic marker in multiple cancers [10, 11]. Interestingly, CIP2A is mainly expressed in the central nervous system, suggesting that CIP2A might strongly correlate with neurological diseases [12]. Since CIP2A is an endogenous inhibitor of PP2A [9], coupled with the fact that decreased PP2A activity could accelerate pathological p-α-syn aggregation, the key feature in PD pathogenesis, it is feasible to explore the roles of CIP2A in PD. Here, we compared the concentration of plasma CIP2A between PD patients and healthy controls and then profiled the expression of CIP2A in classical PD cell and mouse models. In addition, we performed gene silencing studies to assess the effects of the cancer-related gene-CIP2A in the progression of PD.

## Methods

### Participants

The study participants were recruited from the Union Hospital, Tongji Medical College, Huazhong University of Science and Technology. A total of 125 participants were included in this study: 62 patients with PD and 63 healthy controls. Following the MDS Clinical Diagnostic Criteria for Parkinson’s Disease [13], only patients who were clinically diagnosed with idiopathic PD (“diagnosed as PD” or “probable PD”) were included. The healthy controls (*n* = 63) were recruited from medical examination center or family members of the patients. The study conformed with the World Medical Association Declaration of Helsinki, and all human studies were approved by the ethics committee of Tongji Medical College, Huazhong University of Science and Technology. All the subjects gave a written informed consent.

### Clinical Statistics and Plasma Collection

The PD patients underwent evaluation of motor symptoms such as Unified Parkinson’s Disease Rating Scale Part III (UPDRS-III) and non-motor symptoms evolving the Mini-Mental State Examination (MMSE), the Hamilton Depression Scale (HAMD), and the Hamilton Anxiety Scale (HAMA). Specifically, among the 62 PD patients, we evaluated and collected UPDRS-III scores of 34 patients, MMSE scores of 35 patients, HAMD scores of 22 patients, and HAMA scores of 21 patients. Three ml of blood was collected from each participant into purple top EDTA tubes and then centrifuged at 1000 g for 15 min within 12 h. The supernatant was separated, aliquoted, and eventually stored at − 80℃ for < 3 months before testing.

### Enzyme-Linked Immunosorbent Assay (ELISA) Analysis

The stored plasma was thawed on ice. The concentration of CIP2A in the plasma was then evaluated using ELISA kit (Bio-Swamp, Wuhan, China), following the manufacturer’s instruction. The soluble CIP2A levels were estimated twice for each sample. The concentrations were expressed in nanograms per milliliter by reference to a standard curve. The detection range of the ELISA was 0.12 –9.6 ng/ml.

### Antibodies and Reagents

Rotenone and MPTP were purchased from Sigma-Aldrich (R8875, M0896, St. Louis, MO, USA); rabbit polyclonal α-syn phospho-S129 antibody, sheep polyclonal α-syn antibody, and sheep polyclonal tyrosine hydroxylase (TH) antibody were obtained from Abcam (ab51253, ab6162, ab113, UK); rabbit polyclonal PP2A and CIP2A antibodies were from CST (2038 T, 14805S, MA, USA); rabbit polyclonal CIP2A antibody was obtained from ABclonal (A12267, Wuhan, China), while rabbit polyclonal α-syn antibody was purchased from Genetex (GTX112799, San Antonio, TX, USA). siRNA for CIP2A was designed by RiboBio Co., Ltd., Guangzhou, China, as follows: siCIP2A1 CTGTGGTTGTGTTTGCACT; siCIP2A2 GCTCTACTGCGCTGGTTAA; siCIP2A3 CACGGACACTTGCTAGTAT. The activity of PP2A was assayed using a PP2A immunoprecipitation phosphatase assay kit from Sigma-Aldrich (17–313, St. Louis, MO, USA).

### Cell Culture and Transfection

Human SH-SY5Y neuroblastoma cells were obtained from China Center for Type Culture Collection. The cells were cultured and maintained in 90% DMEM/F12 medium (Hyclone, SH30023.01) supplemented with 10% fetal bovine serum (FBS; 11,011–8611, Sijiqing, Hangzhou, China). The cells were then incubated at 37 ℃ in a 5% CO_2_ incubator, and the media was replaced every 2 days. At a confluency of between 30 and 50%, the cells were transfected with relevant three siRNAs or controls by Lipofectamine 2000 (Invitrogen, USA) for 4–6 h without addition of serum and antibiotics. The transfected cells were cultured with normal growth medium for 48–72 h. The CCK-8 assay showed that rotenone (0.25–0.75 uM) was suitable for subsequent experiments with no obvious toxicity.

### PP2A Activity Assays

PP2A activity was performed with cell lysates or mouse brain using the PP2A Immunoprecipitation Phosphatase Assay Kit (17–313, Millipore, Temecula, CA, USA), according to the manufacturer’s instructions.

### Immunocytochemistry

The SH-SY5Y cells were cultured in a 24-well-plate on gelatin-coated glass cover slips. The cells were fixed in 4% paraformaldehyde (PFA) for 10 min and permeabilized with 0.5% Triton X-100 for 10 min. The cells were then blocked in 10% donkey serum for one hour, followed by incubation with the following antibodies: p-α-syn (1:1000), α-syn (1:500), or CIP2A (1:100) for 12 h. After washing with PBST (phosphate-buffered saline containing 0.05% Tween 20), the cells were incubated with secondary antibodies at room temperature for 1 h. Thereafter, the cells were washed in PBST in darkness, followed by incubation with DAPI for 5 min. The cells were washed four times in PBST in the dark before addition of mounting media containing the anti-fade agent and then covered with a coverslip. Images were taken under a fluorescence microscope (Olympus, Tokyo, Japan) or confocal fluorescence microscope (Nickon, Tokyo, Japan).

### Co-immunoprecipitation (Co-IP)

The co-IP analysis was carried out as previously described [14]. We used antibodies against CIP2A, α-syn, or rabbit IgG (A7016, Beyotime Biotechnology, Co., Ltd., China). The agarose affinity gels used for the Co-IP were purchased from Beyotime, P2006. Briefly, the lysed SH-SY5Y cell samples were incubated with the antibodies, and then Protein A beads were added into the mixture and incubated. The beads were washed and solubilized in SDS sample buffer and then assessed by immunoblotting assays.

### Immunoblot

Tissue extracts or cell lysates were prepared, and the concentration was estimated using the BCA protein assay (Beyotime Biotechnology, Co., Ltd., China). The concentration of stacking and resolving gel was selected based on the molecular weight of the target protein. The samples (15 μg) were loaded and separated in the SDS-PAGE gel and then transferred to a PVDF membrane. The blots were blocked in 5% skimmed milk in PBS for 1 h at room temperature, followed by incubation with diluted primary antibodies at 4 ℃ overnight. Thereafter, the blots were incubated with secondary antibodies for 1 h and washed with TBST. The blots were processed with ELC reagents (Servicebio, Wuhan, China), and then the signals were detected under the Bio Rad imaging system. The images were saved for further analysis by Image J software.

### Animals

A total of 45 C57BL/6 male mice aged 8 weeks old were purchased from Beijing Vital River Laboratory Animal Technology Co., Ltd. The animals were kept in a specific pathogen free (SPF) animal house at the Animal Experiment Center of Tongji Medical College of Huazhong University of Science. The mice were kept in a 12-h dark and night cycle with free access to water and food. The mice were acclimatized in the SPF animal room for 2 weeks. All the animal procedures followed the standards of the Animal Care and Use Committee of Huazhong University of Science and Technology.

The C57BL/6 mice were randomly divided into three groups, the control group, the MPTP group and the rotenone group. MPTP (30 mg/kg/d) solution was intraperitoneally administered to the mice continuously for 7 days (from day 1 to day 7), while equal amount of saline was used for the normal control group. The mice were subjected to behavioral tests for 7 days after the last MPTP injection. After the behavioral tests, the mice were decapitated for subsequent histological analysis. Rotenone groups were orally administered rotenone (30 mg/kg) for 8 weeks; after modeling, the mice were decapitated for subsequent histological analysis.

### Behavioral Analysis

To evaluate the MPTP induced behavioral deficits, the control and MPTP treated mice were assessed using rotarod test and pole test (*n* = 8 to 9 mice per group). Briefly, mice were trained for 3 consecutive days before the actual test, and then each mouse was subjected to behavioral tests for 7 days after saline or MPTP injection. Rotarod test: The mice were habituated on the lanes for 2 min, and then rotarod apparatus was set to accelerate from 5 and maintained at 30 rpm for 300 s. The time the mice fell from the lanes was recorded and averaged with the time interval set at 1 min for each mouse to undergo three independent tests. Pole test: A mental rod of 1 cm in diameter and 50 cm in height was wrapped with bandage gauze, and a metal ball (or cork ball) of 25 mm in diameter was placed on the top of the rod. The mice underwent 3 rounds of pre-training with the pole to ensure that all the mice would bow their heads once they were placed on the ball. Then the mice were placed on the ball with the head upside down. The time taken to and from halfway to the base of the pole was recorded. On day 14, the mice were sacrificed.

### Immunohistochemistry (IHC) and Immunofluorescence (IF) Staining

The mice were anesthetized and then underwent transcardial perfusion with saline at a low flow rate for 3 min. Brains of the mice were fixed by 4% PFA through vascular perfusion, dissected, and then immersed with 4% PFA at room temperature for one day. Sections of SN or striatum were cut, dehydrated through gradient alcohol concentration, embedded in paraffin blocks and then sliced on a microtome at the thickness of 3–4 um. According to the previous literature [15, 16], three SN sections per mouse from Bregma − 3.0, − 3.3, and − 3.6 mm were used for TH^+^ cells staining and counting. The sections were floated on water with 40 ℃ before being transferred onto glass slides. Afterwards, the slides were immersed with xylene, subjected to a gradient concentration of alcohol for rehydration, and incubated with 3% H_2_O_2_ to block endogenous peroxidase activity. Lastly, the slides were incubated with the primary antibodies, followed by incubation with biotin conjugated secondary antibody or appropriate fluorescent secondary antibodies conjugated to Alexa-fluor 488, 594 or 647 (Jackson, West Grove, PA, USA). Images were taken under a light or fluorescence microscope (Olympus, Tokyo, Japan).

### Statistical Analysis

The data were analyzed by GraphPad Prism 7.00 software. Shapiro–Wilk normality test was used to validate conformation of the values with Gaussian distribution. Thereafter, the values were compared with unpaired *t* test (expressed as mean ± SEM). For variables that did not meet the assumption of normality or homoscedasticity, the groups were compared with nonparametric Mann–Whitney *U* test, expressed as median (P25%–P75%). Diagnostic accuracy of plasma CIP2A level was assessed with receiver operating characteristic curve (ROC) analyses as well as the identified cutoff values based on the highest Youden J index. Differences between more than two groups were analyzed by a one way-ANOVA, if the statistics complied with homogeneity of variance. Otherwise, nonparametric test was employed. For comparison of constituent ratios such as age, chi-square test was applied. We performed correlation analysis between variables using Spearman correlation or Pearson correlation.

## Results

### CIP2A Concentration Is Decreased in the Plasma of Patients with PD

#### Clinical Characteristics

Out of the 125 individuals included in this study, 62 were PD patients (mean ± SEM age, 62.9 ± 1.321 years), and 63 were healthy controls (mean ± SEM age, 61.19 ± 1.025 years). Demographic and clinical data are shown in Table 1. Our analysis showed that there was no significant difference in terms of age or gender distribution. Besides, the scale information like disease duration, UPDRS-III, MMSE, HAMD, and HAMA of each PD patient was also collected (Table 1). The mean UPDRS-III score for the PD group was 34.5 (18.75–45.75), *n* = 34, Mean MMSE score was 27 (23–28), *n* = 35, and the mean scores of HAMD as well as HAMA were 17.95 ± 2.113 and 18.14 ± 2.466, respectively.

**Table 1:** Demographic and clinical information of the PD and control groups

Items information	PD, *n* = 62	NC, *n* = 63	*p* value
Male, %^a^	41.94	46.03	0.6446
Age, y, mean ± SEM^b^	62.90 ± 1.321	61.19 ± 1.025	0.3068
Duration, y, median (P25%–P75%)^c^	3 (2–7)	―	―
UPDRS, median (P25%–P75%)^c^	34.5 (18.75–45.75), *n* = 34	―	―
MMSE, median (P25%, P75%)^c^	27 (23–28), *n* = 35	―	―
HAMD, mean ± SEM^b^	17.95 ± 2.113, *n* = 22	―	―
HAMA, mean ± SEM^b^	18.14 ± 2.466, *n* = 21	―	―
CIP2A (ng/ml), median (P25–P75%)^c^	1.721 (1.435–2.428)	3.051 (2.36–5.475)	< .0001

^a^Chi‐square test

^b^Unpaired Student’s *t* test

^c^Mann-Whitney *U* test

#### Diagnostic Utility of Plasma CIP2A in PD

**Fig. 1:**
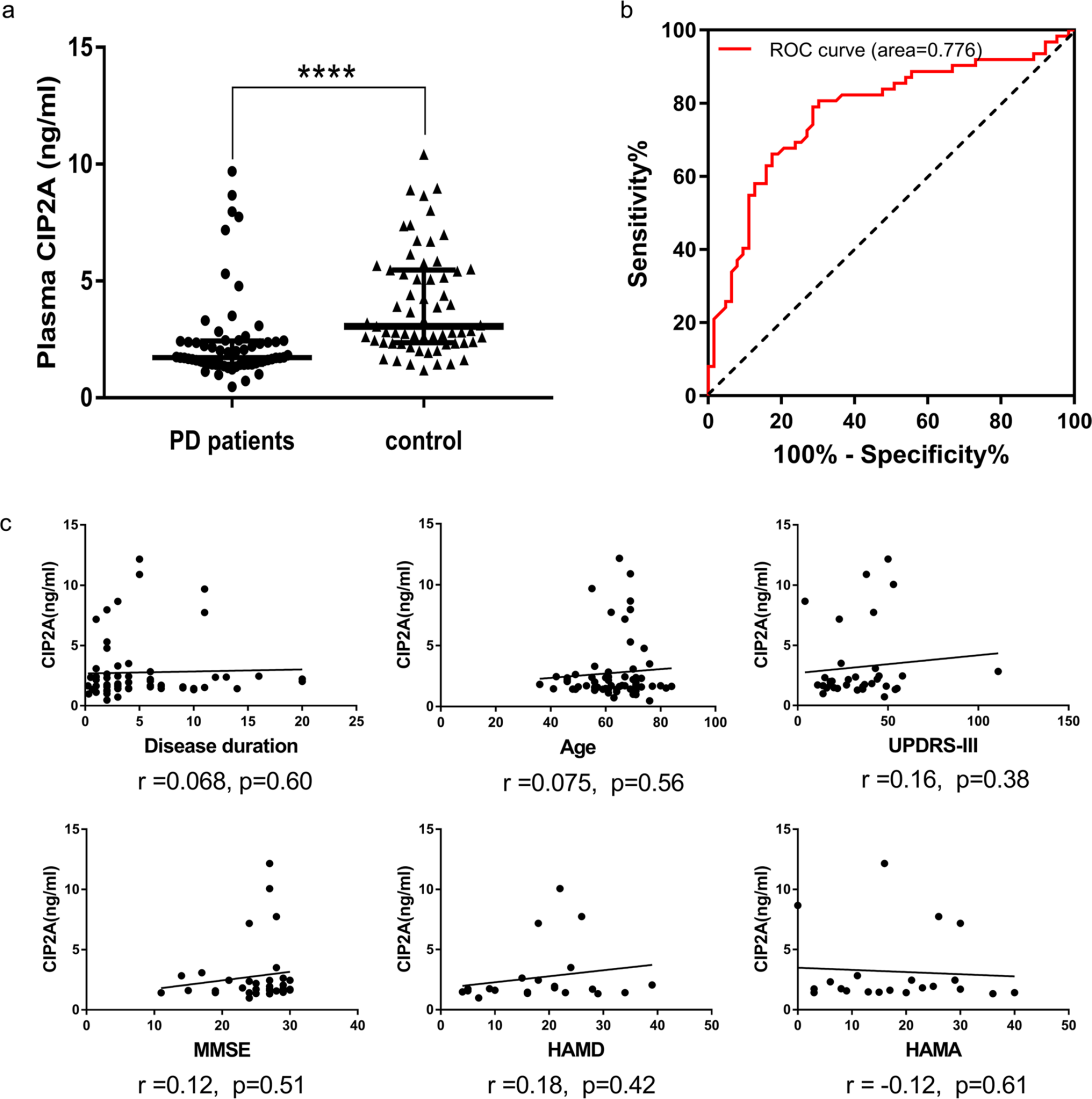
Evaluation of plasma CIP2A concentrations in PD patients and neurological controls. **a** Plasma CIP2A levels were significantly decreased in PD patients as compared to the controls, 1.721 (1.435–2.428) ng/ml vs 3.051 (2.36–5.475) ng/ml, Mann–Whitney *U* test (for non-parametric data), *p* < 0.0001. **b** A ROC curve was generated across all values, and the area under the curve was 0.776, when the cut-off value was set as 2.528 ng/ml, the optimum sensitivity–specificity balance was acquired. **c** Correlation of the plasma CIP2A concentrations with the motor and non-motor symptoms. The plasma CIP2A concentrations did not correlate with disease duration (*r* = 0.06802, *p* = 0.5994), age (*r* = 0.075, *p* = 0.56) or UPDRS III motor scores (*r* = 0.1557, *p* = 0.3792). For the assessment of non-motor symptoms, there was no CIP2A correlation between MMSE scores (*r* = 0.1152, *p* = 0.51), HAMD scores (*r* = 0.181, *p* = 0.4201), and HAMA scores (*r* =  − 0.1189, *p* = 0.6077) in the PD groups. ****p < *0.0001*

To compare and profile the CIP2A expression in the plasma of PD patients and healthy controls, the plasma was collected and the CIP2A concentration was estimated using ELISA. Surprisingly, the concentration of CIP2A was significantly low in the PD groups compared with the healthy controls 1.721 (1.435–2.428) ng/ml vs 3.051 (2.36–5.475) ng/ml, Mann–Whitney *U* test, *p* < 0.0001 (Fig. 1a). To assess whether plasma CIP2A could be used as a biomarker in the diagnosis of PD, the ROC analysis was performed. The data showed that the appropriate cutoff value was 2.528 ng/ml, with a sensitivity of 80.65% and specificity of 69.84%, and the area under the curve was 0.776 (Fig. 1b).

We then assessed the association, if any, between the plasma CIP2A and clinical variables (Fig. 1c). Spearman correlation analyses demonstrated that plasma CIP2A level was marginally correlated with UPDRS III motor scores (*r* = 0.1557, 95% CI − 0.2026 to 0.4773, *p* = 0.3792). Similarly, for non-motor symptoms assessment, there was no significant correlation between CIP2A and MMSE scores (*r* = 0.1152, 95% CI − 0.2365 to 0.4401, *p* = 0.51), HAMD scores (*r* = 0.181, 95% CI − 0.2728 to 0.569, *p* = 0.4201), and HAMA scores (*r* =  − 0.1189, 95% CI − 0.5336 to 0.3418, *p* = 0.6077) in the PD groups. In addition, the plasma CIP2A seemed not correlated with disease duration (*r* = 0.06802, 95% CI − 0.1922 to 0.3193, *p* = 0.5994) and age (*r* = 0.075, 95% CI − 0.1784 to 0.3185, *p* = 0.56, Pearson correlation analyses). In general, the plasma CIP2A could be a potential new PD biomarker despite lack of positive correlation with disease severity.

#### Overexpression of CIP2A in PD Cell Model

Because of the ambiguous role of CIP2A in the PD pathogenesis, we evaluated the expression of CIP2A in PD cell model. CCK-8 testing showed that the viability of SH-SY5Y cells exposed to different concentrations of rotenone (0.25 μM, 0.5 μM, 0.75 μM, and 1.0 μM) decreased from 84.35%, 82.74%, 78.89%, and 41.00% after 24 h. Based on these findings, we selected 0.25 uM and 0.5 uM of rotenone and added them into SH-SY5Y cells for 24 h to induce the PD cell model.

**Fig. 2:**
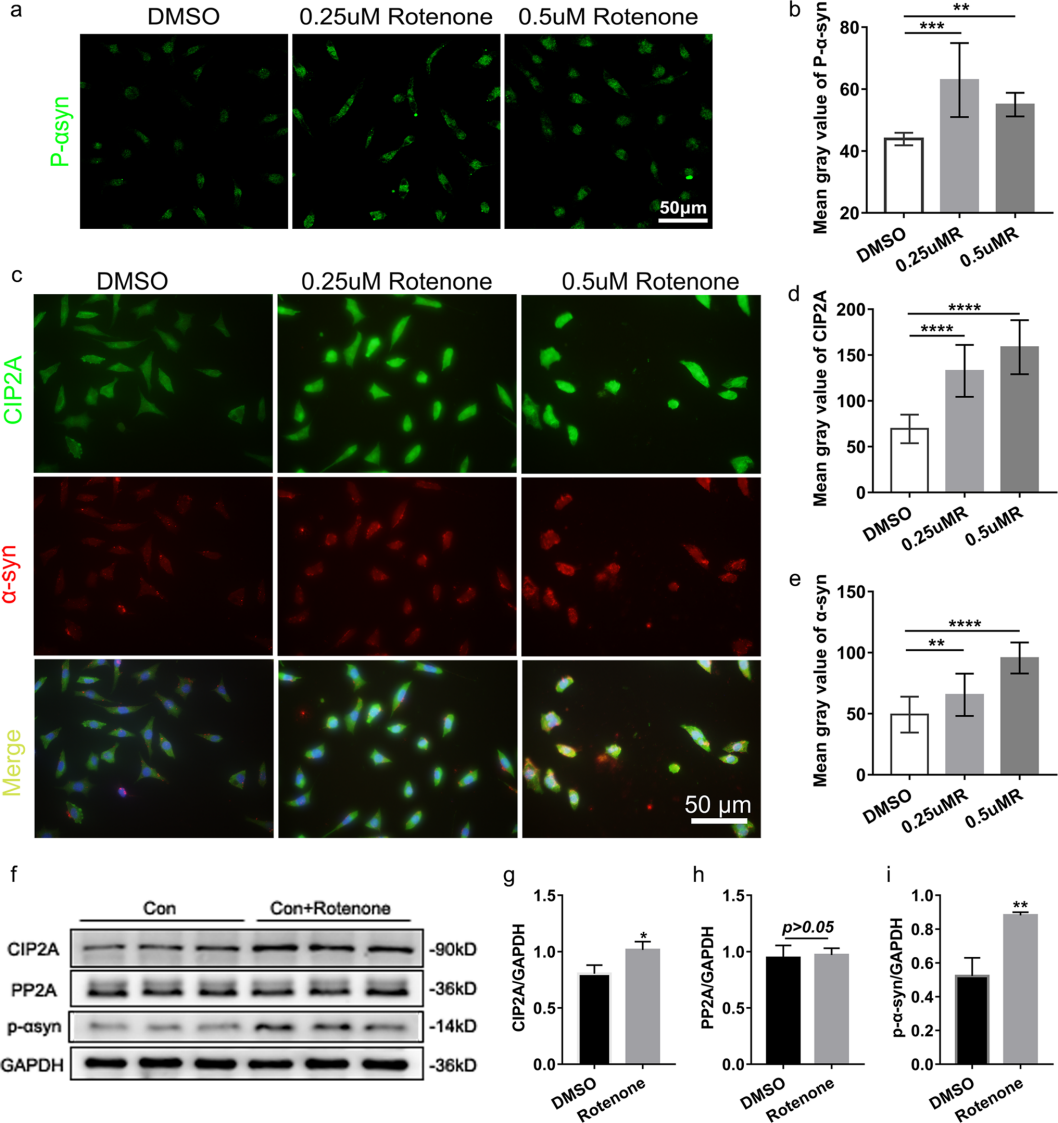
Assessment of CIP2A expression levels in rotenone treated PD cell models. **a, b** The IF staining of p-α-syn (green) increased following incubation of the SH-SY5Y cells with 0.25 uM and 0.5 uM rotenone for 24 h. **c–e** IF staining showed increased CIP2A (green) expression in PD group and colocalized of α-syn (red) with CIP2A. **f** Western blotting results of CIP2A**,** PP2A, and p-α-syn revealed the ascending CIP2A and p-α-syn after rotenone treatment; β-actin served as the loading control. **g–i** Quantification of CIP2A/GAPDH, PP2A/GAPDH, or p-α-syn/GAPDH ratio, the bars represent the mean ± SEM. Histograms summarize the mean IF signal intensity ± SEM as measured in gray values. *p < 0.05, **p < 0.01, ***p < 0.001, ****p < *0.0001*

As expected, the IF assay demonstrated significant upregulation of p-α-syn after treatment with rotenone (Fig. 2a, b), which were confirmed by western blotting analysis (Fig. 2f, i). Besides, the IF data showed that the CIP2A expression was colocalized with α-syn after rotenone treatment (Fig. 2c–e). Immunoblot assays confirmed the overexpression of the CIP2A and p-α-syn in the PD group, while no significant change was observed in the expression of PP2A (Fig. 2f–i). Importantly, overexpression of the CIP2A in the PD cell model diminished the PP2A activity, as evaluated by dephosphorylation of the phosphopeptide (K-R-pT-I-R-R) (Fig. 4i). These results taken together indicated that CIP2A expression levels were increased in PD cell models, leading to the reduced activity of PP2A.

#### Increased Expression of CIP2A in Mouse Models of PD

**Fig. 3:**
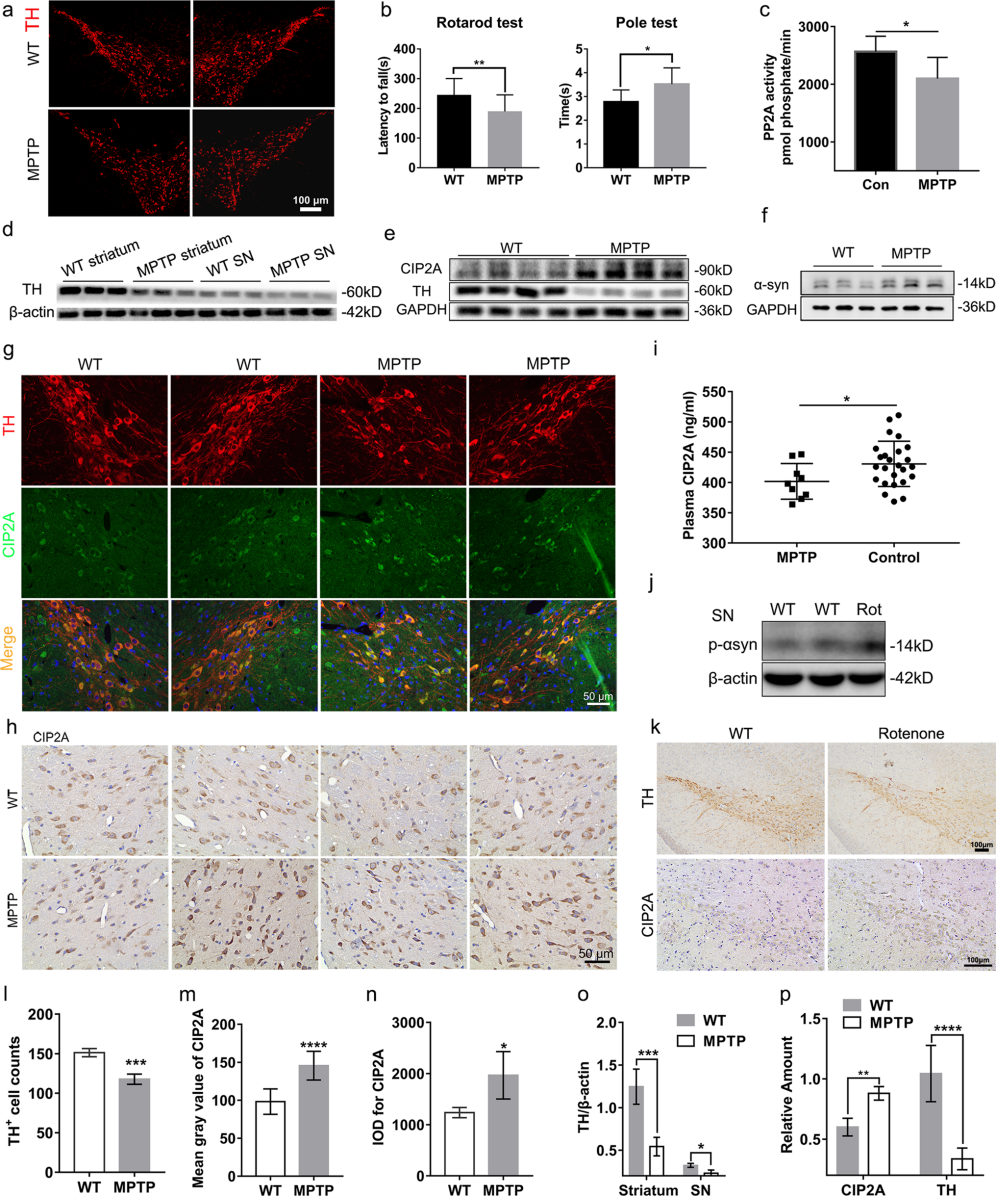
Evaluation of CIP2A expression in MPTP induced subacute mouse models. **a, l** Representative IF images and the quantification of the number of TH + neurons in the SN at 14 days after treatment with MPTP, *n* = 4 to 6 brains per group. **b** Behavioral test of the mice showed decreased time in rotarod test and extended time in pole test after MPTP treatment. Data were expressed as means ± SEM, *n* = 8 to 9 mice per group. **c** Compared with the control group, there was suppression of the PP2A activity of the brain tissue of the MPTP-treated mice, *n* = 6 brains per group. **d**, **o** Immunoblot results demonstrating the decreased TH expression in both the SN and striatum of the MPTP-treated mice; β-actin served as the loading control. **e**, **p** Immunoblot results demonstrating CIP2A expression in the SN of the mice. **f** Immunoblotting of α-syn**. g**, **h**, **m**, **n** IF staining of CIP2A (green) and TH (red) as well as IHC assay showed increased CIP2A expression in the SN after MPTP treatment, *n* = 4 to 6 brains per group. **i** Plasma CIP2A levels were significantly decreased in the MPTP treated mice when compared to those in the controls. **j** Immunoblotting of p-α-syn in the SN of rotenone model. **k** IHC staining of TH and CIP2A in the SN of rotenone model. Histograms summarize the mean IF signal intensity detected ± SEM as measured in gray values. *p < 0.05, **p < 0.01, ***p < 0.001, ****p < *0.0001*

We then assessed if above pathological changes applied to the PD mouse models. The subacute PD mouse model was induced by intraperitoneal injection of MPTP (30 mg/kg/d) for 7 consecutive days [17]. Behavioral tests by rotarod and pole experiments (Fig. 3b) showed that the MPTP treated groups induced movement defects inferred from the relatively shorter time in rotarod test and longer time in pole test, consistent with previous studies [18, 19]. Moreover, we found that there were fewer TH^+^ dopaminergic neurons in the similar level sections of SN in MPTP group than control group (Fig. 3a and l). The reduction of TH was further quantified by western blotting of the SN and striatal lysates (Fig. 3d and o), while the level of α-syn was increased in MPTP group (Fig. 3f), thus indicating a successful establishment of a PD mouse model.

We then evaluated the concentration of CIP2A in plasma of PD mouse model in comparison with the control group. Consistent with the data from the PD patients, the CIP2A concentration was significantly lower in the MPTP treated group (Fig. 3i). In addition, the CIP2A expression levels were examined in the SN of the mice models. Consistent with cellular results, immunoblot analysis showed increased expression of CIP2A in the MPTP treated mice (Fig. 3e and p). This result was further validated by IHC and IF analyses (Fig. 3g, h, m, and n). We next examined the activity of PP2A in the brain, which theoretically inversely correlate with CIP2A. As expected, there was suppression of the activity of PP2A (Fig. 3c). We demonstrated that the expression of CIP2A in intracranial lesions was increased but decreased in peripheral blood.

To further assess the expression of the CIP2A, we used oral rotenone (30 mg/kg) administration to model PD. The TH expression level was decreased (Fig. 3k), while the p-α-syn level was increased (Fig. 3j), indicating that the model was successfully established. Consistent with the results of the MPTP model, IHC showed increased expression of CIP2A in mice treated with rotenone (Fig. 3k).

#### Knockdown of CIP2A Expression Reduces the PD-Related Pathology

**Fig. 4:**
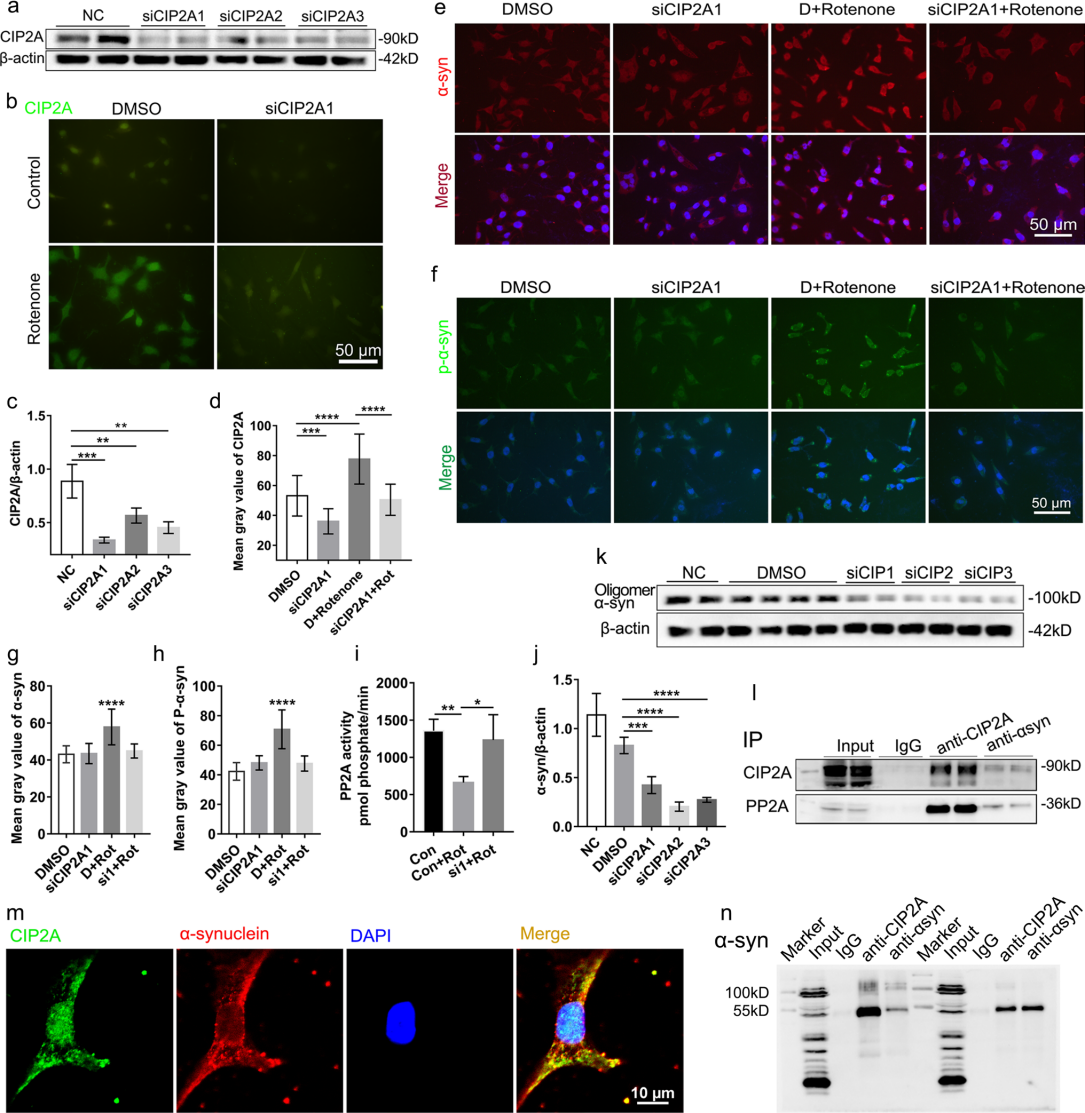
Assessment of the effect of CIP2A knockdown on rotenone induced PD pathology. **a**, **c** Western blotting assays were used to evaluate the interference effects of the three siRNAs on the CIP2A expression. **b**,** d** IF staining of CIP2A (green) confirmed the effect of CIP2A knockdown by siCIP2A1. **e**, **f**, **g**, **h** Representative IF staining of the p-α-syn and α-syn in the control, siRNA-treated, rotenone-treated, or siRNA + rotenone-treated group. **i** The PP2A activity was decreased after rotenone exposure but increased after siCIP2A treatment. **k, j** The α-syn expression decreased after treatment with the three pairs of CIP2A siRNAs. **l**,** n** CIP2A interacts directly with α-syn. Co-IP and western blot analysis of the CIP2A and α-syn from the SH-SY5Y cells; normal rabbit IgG served as the controls. **m** Confocal fluorescence microscopy showing colocalization of CIP2A and α-syn. Values are given as means ± SEM. Statistical significance was determined by one-way ANOVA. Histograms summarize the mean IF signal intensity detected ± SEM as measured in gray values. *p < 0.05, **p < 0.01, ***p < 0.001, ****p < *0.0001*

We then explore whether manipulating the level of CIP2A would affect the expression of pathological α-syn, the main feature of PD. siRNA was used to suppress the CIP2A expression in SH-SY5Y cells. We first evaluated the knockdown effect on oligomeric α-syn expression, an extensively reported form of the pathological α-syn, and demonstrated that all three treated siRNA substantially reduced the oligomeric α-syn (Fig. 4k, j). Simultaneously, the efficiency of the 3 different CIP2A siRNAs was assessed by immunoblot and IF assays, and the data showed that siCIP2A1 had the highest efficiency (Fig. 4a–d). The siCIP2A1 was then used for subsequent experiments. We demonstrated that the expression of the other pathological forms of α-syn, p-α-syn (ser129), and total α-syn was obviously decreased after CIP2A silencing in the rotenone-induced PD cell model (Fig. 4e–h). Besides, the PP2A activity decreased significantly in rotenone-induced SH-SY5Y cell model simulating PD, but no obvious change after knockdown of CIP2A in cells without rotenone treatment (Fig. 4i). Thus, these data implicate the role of PP2A in the pathogenesis of PD. Hence, reduced CIP2A could interfere with the rotenone-specific pathology, which might result from the enhancement of PP2A activity [5, 6].

We further verified the decreased plasma CIP2A levels in the PD patients using PD mouse model, which was contrary to the increased expression in the PD cell and mouse model. Our confocal fluorescence microscopy analysis showed colocalization of the CIP2A and α-syn (Fig. 4m), an observation confirmed by endogenous CIP2A immunoprecipitation, where some PP2A was also co-immunoprecipitated (Fig. 4l). Besides, Co-IP and western blot analyses of the CIP2A and α-syn from the SH-SY5Y cells showed that CIP2A might directly interact with α-syn (Fig. 4n). Therefore, we speculated that the increased CIP2A binding with α-syn in the lesioned areas of the brain, suppressed the release of CIP2A to the peripheral blood.

## Discussion

As PD is featured by a long preclinical phase, objective biomarkers have significant clinical value in monitoring disease development [20]. Here, we identified and attempted to validate CIP2A as a potential novel biomarker in PD. Previous studies focused on the α-syn, the key molecule in PD pathogenesis, in which the total, oligomeric, or phosphorylated forms were investigated in the blood or cerebrospinal fluid (CSF) [21–23]. Although the most prominent biomarker evaluation relied on the level of different α-syn types, its sensitivity and specificity have been suboptimal. Considering that lumbar puncture is inevitable for CSF acquisition and the relative difficulty associated with the procedure, CSF is not an ideal biomarker candidate. Blood samples are more readily available and might be more promising sources of biomarkers. However, the concentration of α-syn in blood is influenced by the contamination and hemolysis of red blood cells, a major source of α-syn in blood, thus explaining the inconsistent and sometime controversial results. Other blood biomarkers include DJ-1, uric acid, epidermal growth factor, apolipoprotein-A1, uMtCK activity, and peripheral inflammatory markers [24–27]. However, few studies focused on early disease stage [28] and lack of ideal biomarkers for diagnostic sensitivity and specificity persists.

In our study, we demonstrated that CIP2A declined in PD patients with different disease durations (early, medial, and late stages of development) and might be a promising candidate in the PD diagnosis based on its specificity, sensitivity, and availability. Since CIP2A inhibits PP2A activity and could promote the α-syn phosphorylation at ser-129 locus, CIP2A might reflect the p-α-syn changes. Therefore, there could be CIP2A expression shifts associated with the emergence of Parkinsonian pathological changes, huge deposition of p-α-syn in the brain, with no substantial variation in later clinical stages for PD. Hence, the CIP2A level might not substantially vary with PD progression, which might be the underlying cause for weak correlation between the CIP2A concentration and disease progression as well as motor and non-motor symptoms.

We then showed upregulation of CIP2A in PD cell and animal models. The pathological p-α-syn accumulation in the SN or cortex was strongly associated with reduced activity of PP2A [8, 29, 30]. We next demonstrated that CIP2A participated in the pathogenesis of PD and that knockdown of CIP2A restored PP2A activity and reduced the levels of p-α-syn. Presumably, this protective effect was due to direct action of the increased activity of PP2A as revealed in our assays, leading to degradation of the phosphorylated α-syn [6, 7].

Besides, we showed that there was decrease in the level of plasma CIP2A but high expression in the brains of PD mouse and cell models. Physiologically, CIP2A is expressed at a very low level in most non-malignant tissues [31], but it is enriched in the brain. Sequence analysis of the CIP2A protein showed it has multiple protein interaction regions [9]. For instance, CIP2A could inhibit PP2A activity by directly binding to c-myc with ser-62 site in human malignancies (head and neck squamous cell carcinoma or colon cancer) [9]. Therefore, we speculated that the increased binding of α-syn with CIP2A (Fig. 4l, n) in the brain might induce low expression in plasma. This is also consistent with the status of the α-syn in the PD patients. Several studies have observed that, compared with controls, the concentration of α­-syn in CSF is decreased in PD patients, while the α-syn level in the brain tissue of the PD patients is upregulated [23, 32]. The reduction of CIP2A in plasma of PD patients is likely because of α-syn as well as CIP2A aggregation and sequestration in LBs, similar to the reduction of α-syn in CSF. Therefore, it is important to measure the level of CIP2A in post-mortem PD brains.

This study is limited by the relatively small sample size, especially in evaluating data such as the scales of motor and non-motor symptoms. There is need for further clinical studies involving a larger number of patients to acquire a more accurate conclusion. Besides, there is need for a longitudinal study to evaluate the dynamic change of CIP2A in a single patient during disease progression as well as a definite site of CIP2A interaction with α-syn.

Taken together, our data showed that the plasma CIP2A concentration is significantly lower in the PD patients compared to healthy controls. Besides, the involvement of CIP2A in PD cell and animal models and the findings that CIP2A silencing could alleviate PD pathogenesis confirmed the role of CIP2A in PD. Thus, the CIP2A might be a novel potential diagnostic biomarker and therapeutic target for PD.

## Data Availability

All data included in this study are available upon request by contact with the corresponding author.
